# Effect of preoperative immunonutrition on outcomes of colon cancer surgery: study protocol for a randomized controlled trial

**DOI:** 10.1186/s13063-020-04544-3

**Published:** 2020-07-08

**Authors:** Soo Young Lee, Seung-Seop Yeom, Chang Hyun Kim, Hyeong Rok Kim

**Affiliations:** Department of Surgery, Chonnam National University Hwasun Hospital and Medical School, 322 Seoyang-ro Hwasun-eup, Hwasun-gun, Jeonnam 58128 South Korea

**Keywords:** Colon cancer, Immunonutrition, Complication, Infection, Randomized controlled trial

## Abstract

**Background:**

Current guidelines recommend the prescription of immune-enriched oral nutritional supplements for malnourished patients before major gastrointestinal surgery. However, the benefit of preoperative immunonutrition is still controversial. This randomized controlled trial aims to evaluate the effect of preoperative immunonutrition on the outcomes of surgery for colon cancer.

**Methods/design:**

Patients with primary colon cancer will be included as study participants after screening. They will be randomly assigned (in a ratio of 1:1) to receive preoperative immunonutrition added to the normal diet (experimental arm) or consume normal diet alone (control arm). Patients in the experimental arm will receive oral supplementation (400 mL/day) with arginine and ω-3 fatty acids for 7 days before elective surgery. The primary endpoint is the rate of infectious complications, while the secondary endpoints are postoperative complication rate, change in body weight, length of hospital stay, and nature of fecal microbiome. The authors hypothesize that the rate of infectious complications would be 13% in the experimental arm and 30% in the control arm. With a two-sided alpha of 0.05 and a power of 0.8, the sample size is calculated as 176 patients (88 per arm).

**Discussion:**

Although there have been many studies demonstrating significant benefits of preoperative immunonutrition, these were limited by a small sample size and potential publication bias. Despite the recommendation of immunonutrition before surgery in nutritional guidelines, its role in reduction of rate of infectious complications is still controversial. This trial is expected to provide evidence for the benefits of administration of preoperative immunonutrition in patients with colon cancer.

**Trial registration:**

Clinical Research Information Service KCT0003770. Registered on 15 April 2019.

## Introduction

Even in the current laparoscopic era, postoperative infectious complications still remain the major concern after colorectal surgery. Surgical stress may cause immunosuppression, resulting in postoperative inflammatory and infectious complications [1]. Rapid recovery of patients in the postoperative period is expected in the absence of complications. Particularly, in surgery for colorectal cancer, postoperative infectious complications, such as anastomotic leakage, are significantly unfavorable because these may lead to omission of adjuvant chemotherapy and poorer oncologic outcomes.

Nutrition plays a key role in recovery after major gastrointestinal surgeries. The nutritional risk rate of cancer patients was reported to be 26 to 76%, higher than that of general patients [2]. Patients with colorectal cancer, especially those with advanced tumor, may have a high nutritional risk due to various symptoms such as anorexia, diarrhea, and intestinal obstruction [3]. Malnutrition is closely related to the clinical results of surgical patients, because it can damage the immune system and wound healing process and degrade respiratory and cardiac functions [2, 4].

Proper perioperative nutritional support may enhance immune function and therefore reduce postoperative complications [1]. In recent years, an enteral diet enriched with immunonutrients such as arginine, ω-3 fatty acids, and glutamine has been used. The present international guidelines recommend that immunonutrition should be given to nutritionally high-risk patients before major oncologic surgeries [5]. These guidelines are based on randomized controlled trials demonstrating a reduction in the rate of infectious complications following the use of preoperative immunonutrition [6]. However, since these randomized controlled trials have been criticized for potential conflicts of interests and the small sample size, the usefulness of preoperative immunonutrition still remains controversial [6].

Therefore, we designed this randomized controlled trial to investigate the effect of preoperative immunonutrition on outcomes of surgery in patients with colon cancer.

## Methods/design

### Study design

This study is a randomized controlled trial investigating the effect of preoperative immunonutrition on outcomes of surgery for colon cancer. This protocol is written in accordance with the Standard Protocol Items: Recommendations for Interventional Trials (SPIRIT) checklist [7].

### Objectives and hypotheses of the study

The primary objective of the present study is to evaluate the impact of preoperative immunonutrition on the rate of postoperative infectious complications in patients with primary colon cancer. As a secondary objective, the study will also assess the relationship between the administration of preoperative immunonutrition and postoperative complication rate, change in body weight, length of hospital stay, and nature of fecal microbiome.

### Study participants

**Fig. 1:**
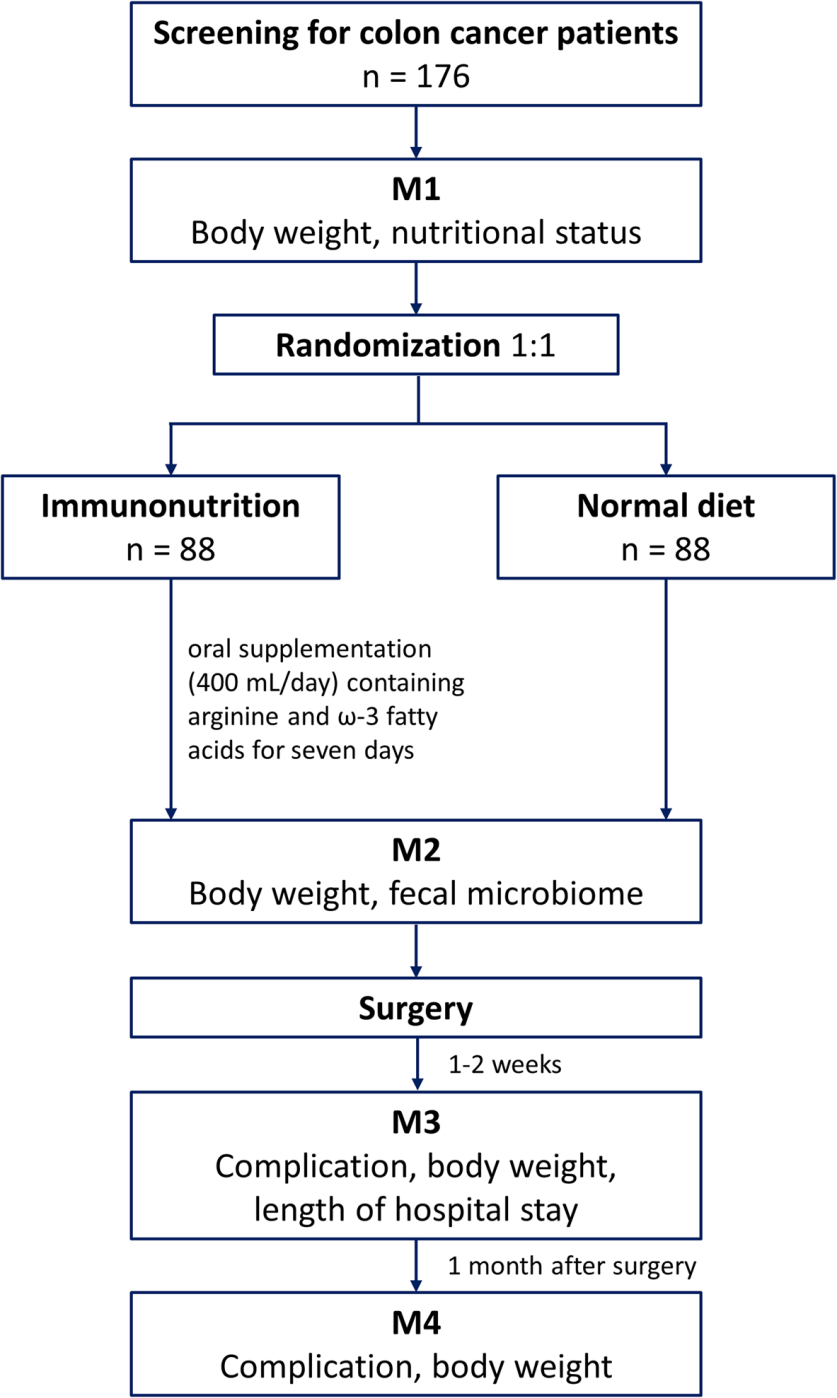
All enrolled patients will be randomized in a 1:1 ratio into one of the following arms after screening: experimental arm (patients who receive preoperative immunonutrition added to the normal diet) or control arm (patients who receive a normal diet alone)

To prove the benefit of immunonutrition, we will enroll primary colon cancer patients who are scheduled to undergo surgical resection. The inclusion criteria will consist of patients with primary colon cancer, patients aged 20–80 years, and those who provide a written consent to participate in the study. The exclusion criteria will consist of patients who need emergency surgery, patients with difficulty in oral intake, pregnant women, and patients planned for ostomy surgery. All enrolled patients will be randomized in a 1:1 ratio into one of the following arms after screening: experimental arm (patients who receive preoperative immunonutrition added to the normal diet) or control arm (patients who receive a normal diet alone) (Fig. 1). Because our institution is a regional cancer center that performs about 300 cases of elective colon cancer surgery annually, we expect that registration will be completed in about a year and a half.

### Immunonutrition

Patients in the experimental arm will receive 400 mL/day of preoperative oral supplementation in the form of an immunonutrient-enriched enteral feed (Newcare Omega®) added to the normal diet for seven consecutive days prior to surgery. Newcare Omega® contains high protein, arginine, and ω-3 fatty acids. The detailed composition of the oral supplementation is shown in Table 1. Since the time from randomization to surgery will vary from participant to participant, the start date will be specified so that oral supplements can be taken equally for 1 week prior to surgery.

**Table 1:** Composition of oral supplementation

	Amount (per 100 mL)
Energy (kcal)	100
Protein (g)	5.0
Fat (g)	3.0
Carbohydrate (g)	13.75
Arginine (g)	0.25
ω-3 fatty acids (EPA + DHA + ALA)	0.23

*EPA* eicosapentaenoic acid, *DHA* docosahexaenoic acid, *ALA* α-linolenic acid

### Ethics and trial registration

All the participants will be completely informed about the protocol of the study, and a written informed consent will be obtained. The protocol of this study was approved by the Institutional Review Board of Chonnam National University Hwasun Hospital (IRB No. CNUHH-2019-062). This clinical trial was registered in the Clinical Research Information Service (CRIS registration No. KCT0003770; https://cris.nih.go.kr/cris/en/).

### Sample size calculation and randomization

Based on a previous meta-analysis, we hypothesize that the rate of postoperative infectious complications would be 13% in the experimental arm and 30% in the control arm [8]. With a two-sided *α* of 0.05 and a power of 0.8, the sample size is calculated as 176 patients (88 per arm). Since the present study compares only short-term outcomes and is very unlikely to cause inconvenience to participants, we think there will be few dropout and do not consider it in calculating sample size. The formula for calculating sample size is as follows.

$$ n=\frac{{\left({z}_{\alpha}\kern0.5em +\kern0.5em {z}_{\beta /2}\right)}^2}{{\left(\delta -\left|\varepsilon \right|\right)}^2}\left[\frac{p_1\left(1-{p}_1\right)}{k}+\kern0.5em {p}_2\left(1-{p}_2\right)\right] $$

$$ \left(\alpha =\mathrm{alpha},\beta =1-\mathrm{power},\varepsilon ={p}_1-{p}_2,\delta =\mathrm{non}-\mathrm{inferiority}\ \mathrm{margin},{n}_1={kn}_2\right) $$

The random assignment will be performed in a 1:1 ratio using computer-generated random numbers. To minimize predictability and selection bias, we will use the maximal procedure for random allocation [9]. Investigators will contact a central randomization center by telephone after the patients are enrolled. Except for one researcher who will be aware of the arm assignment and who will prescribe the oral nutritional supplements, all surgeons and other investigators will be blinded. Owing to the nature of the study, the patients will not be blinded.

### Management and assessment

All participants will receive a second-generation cephalosporin as a prophylactic antibiotic and anti-thrombotic prophylaxis with compression stockings. All patients will undergo surgical resection by a colorectal surgical specialist according to oncological principles, via either a laparoscopic or open approach. Oral intake will be initiated the day after surgery unless obstructive symptoms are reported. The timing of discharge from hospital will be determined by the surgeon, considering achievement of pain control, tolerance of diet, and independent ambulation.

Postoperative complications during 30 days following surgery will be documented and divided into infectious and non-infectious complications. Infectious complications include incisional and organ/space surgical site infection and other infections such as respiratory and urinary tract. Body weight will be recorded at the following four instances: at the time of allocation, the day before surgery, 1–2 weeks after surgery, and 1 month after surgery. A fecal microbiome test will be conducted on 32 consecutive patients among all participants. Samples for the fecal microbiome test will be collected in the hospital a day before the surgery. The detailed schedule of assessments is depicted in Table 2.

**Table 2:**
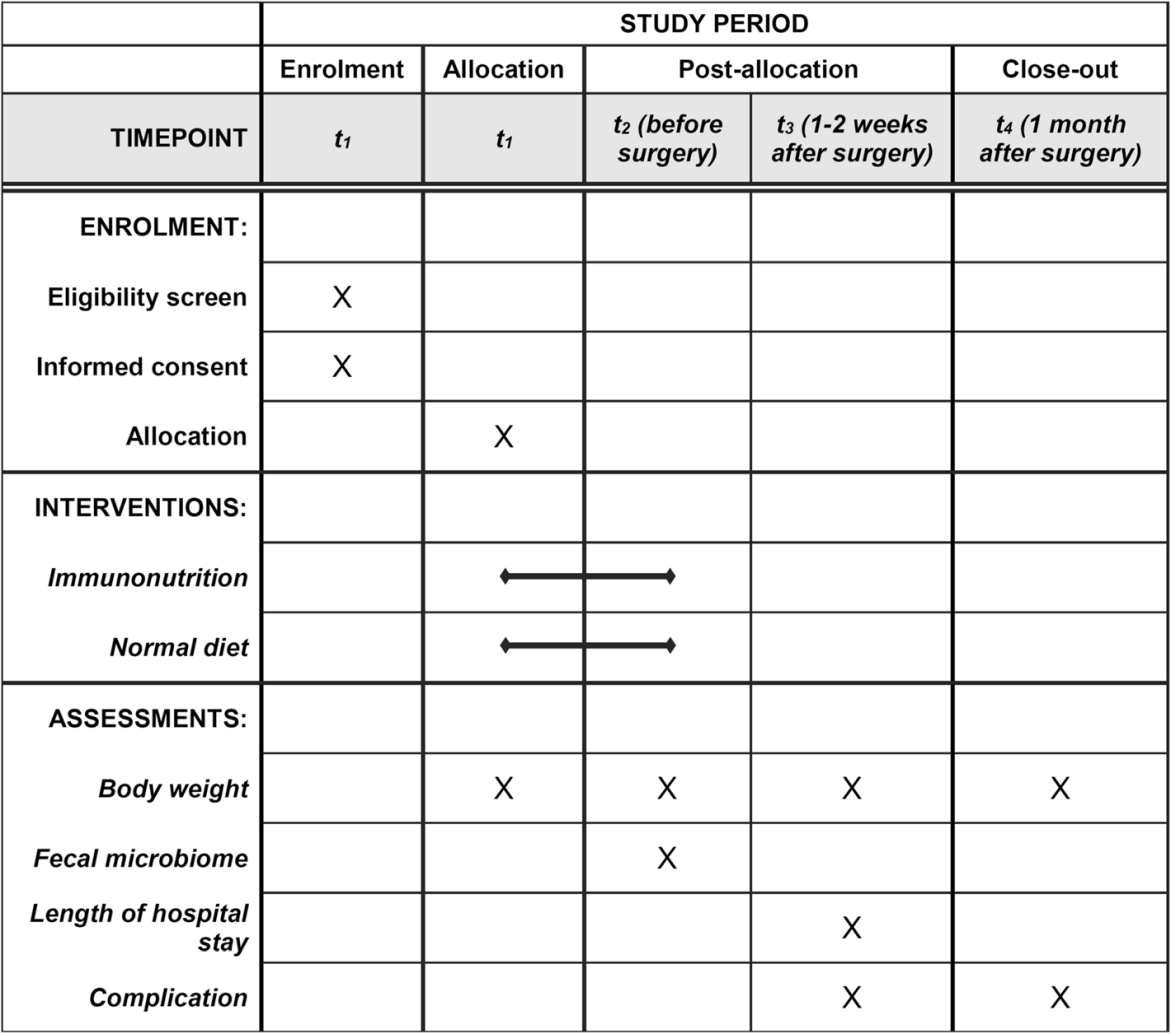
Schedule of assessments

### Fecal microbiome

A total of 32 feces samples will be collected from 16 patients in each group. We will use the QIAamp DNA Stool MiniKit (Qiagen®, Hilden, Germany) to extract genomic DNA from bacteria in the feces, in accordance with the manufacturer’s instructions. Fecal samples will be mixed with 1.4 mL ASL buffer in a 2-mL microcentrifuge tube and will be vortexed. After centrifuging, a 1.2-mL aliquot of the supernatant will be transferred to a new tube, vortexed with an InhibitEX tablet, and centrifuged. The supernatant will be transferred to a new microcentrifuge tube, mixed with 200 μL AL buffer and 15 μL proteinase K and vortexed, and then centrifuged.

The library preparation will be performed following the 16S Metagenomic Sequencing Library Preparation Illumina Protocol. The V3-V4 region of the 16S rRNA will be amplified. Polymerase chain reaction (PCR) will be performed in a total volume of 25 μL containing 2.5 μL of genomic DNA, 5 μL of each primer, and 12.5 μL of KAPA HiFi Hotstart readyMix. PCR will be conducted by performing 25 cycles of denaturing at 95 °C for 30 s, annealing at 55 °C for 30 s, and elongation at 72 °C for 30 s. Following the completion of the first PCR, DNA electrophoresis will be performed and a single amplification product will be checked to verify correct amplification of V4 of 16S rRNA; subsequently, the first clean-up will be performed. The clean-up procedure will be conducted using AMPure XP beads. The second PCR, called index PCR, will be conducted by performing 8 cycles of denaturing at 95 °C for 30 s, annealing at 55 °C for 30 s, and elongation at 72 °C for 30 s. Following the clean-up procedure, the quality of the final library will be checked, and Agilent Bioanalyzer 1000 and Qbit will be utilized to quantify the amount of DNA.

Metagenome will be analyzed using EZBioCloud (ChunLab, Inc.) and BaseSpace (Illumina) platform. Microbial diversity and evenness will be analyzed using Shannon diversity index and Simpson index, and beta diversity will be utilized to identify the correlation between the samples.

### Statistical analysis

Categorical variables will be compared using the *χ*^2^ test or Fisher’s exact test, and continuous variables will be compared using Student’s *t* test. Two-way repeated measures analysis of variance (ANOVA) will be used to compare the change in body weight between the experimental and control arms. Logistic regression will be performed to conduct multivariable analysis. All results will be considered significant at a *p* value of < 0.05. Statistical analyses will be performed using SPSS version 23.0 (IBM Inc., Armonk, NY, USA).

### Data monitoring

A committee will be organized for trial supervision. The entire process of data collection, recording, and management will be monitored by them. The committee may recommend and request the principal investigator to make some changes to the plan.

### Safety evaluation and reporting of adverse event

Adverse events and serious adverse events should be reported to protect the patient. Serious adverse events which could result in death or life-threatening will be reported within 24 h from the detection by investigators. If there are any adverse events caused by the treatment of the clinical trial, compensation shall be made to the participants by our institution according to the prescribed rules.

### Protocol amendments

If needed, the protocol may be modified by communication and agreement of the principal investigator and trial participants. Revised protocol will be approved by the Institutional Review Board.

### Confidentiality

All study-related information will be safely stored at the study site. All participants’ information will be stored in a locked file cabinet in an area with limited access.

### Reporting of the study results

The results of the study will be released to the participating investigators and patients. The study results will be published regardless of the magnitude or direction of effect.

## Discussion

Infectious complications occur at a relatively high rate (14–27%) following intestinal surgery [8]. Among them, anastomotic leakage is one of the most important infectious complications after surgery for colorectal cancer, which may result in compromised oncologic outcomes. We previously reported that a high nutritional risk screening score was associated with anastomotic leakage after surgery for rectal cancer in our institution [2]. Nutritionally high-risk patients were at two-fold (odds ratio [OR], 2.044; 95% confidence interval [CI], 1.085–3.851; *p* = 0.027) higher risk of anastomotic leakage [2]. Since nutritional risk may be improved by adequate preoperative nutritional support, it may play an important role in reducing postoperative infectious complications including anastomotic leakage.

A few previous studies have reported the benefits of preoperative immunonutrition [10–18]. Regarding colorectal cancer, Moya et al. [18] reported that perioperative use of immunonutrition was associated with decreased wound infection. A recent meta-analysis demonstrated that preoperative immunonutrition was associated with a lower rate of infectious complications compared with no oral nutritional supplementation (OR, 0.46; 95% CI, 0.30–0.83; *p* < 0.01) [19]. Based on this result, current guidelines recommend immunonutrition to be provided to malnourished patients preoperatively [5].

However, despite several previous randomized trials, the number of high-quality trials with a low risk of bias is very small [20]. Furthermore, each of the trials had significant shortcomings such as small sample size [12–15, 17] and possible conflicts of interests [10, 11]. The most notable issue is that industry-funded trials reported a positive effect of immunonutrition [10, 11], whereas a non-industry-funded trial with a large sample size showed no clear advantage of preoperative immunonutrition [16]. These conflicting results suggest that publication bias, which implies a tendency to withhold negative results of trials, may be present [20]. A recent meta-analysis reported that some of the measured significant effects were diminished if the biased trials were excluded [20].

The present well-designed, non-industry-funded, randomized controlled trial is an investigator-initiated study that is free from conflicts of interest. In addition, we will recruit a sufficient number of participants (*n* = 176) to obtain reliable results. Another strength of this study is that we attempt to identify the relationship between immunonutrition and fecal microbiome. In colorectal cancer, the role of microbiome in carcinogenesis and postoperative complications has been studied in various ways, and a recent review suggested that malnutrition may be associated with the deterioration of intestinal microbiome, resulting in a higher incidence of postoperative complications [21]. Through this study, we anticipate to arrive at a more definite conclusion regarding the effect of preoperative immunonutrition in patients undergoing surgery for colon cancer.

## Trial status

This trial is in the enrolment stage.

The protocol was registered on CRIS (https://cris.nih.go.kr/cris/en/, KCT0003770) on April 15, 2019.

Protocol version 1.3, December 23, 2019.

Recruitment began on April 17, 2019.

Recruitment is expected to be completed in August 2020.

## Data Availability

Not applicable.
